# Assessing the associations between known genetic variants and substance use in people with HIV in the United States

**DOI:** 10.1371/journal.pone.0292068

**Published:** 2023-10-05

**Authors:** Cameron B. Haas, Kristina M. Jordahl, Robin M. Nance, Bridget M. Whitney, Lu Wang, Joseph A. C. Delaney, Stephanie Ruderman, Tongqiu Jia, Wm. Christopher Mathews, Michael S. Saag, Sulggi A. Lee, Sonia Napravnik, Jeffrey M. Jacobson, Geetanjali Chander, Elizabeth M. McCall, Richard D. Moore, Kenneth H. Mayer, Shubhabrata Mukherjee, Won Jun Lee, Paul K. Crane, Heidi Crane, Inga Peter, Sara Lindström

**Affiliations:** 1 Department of Epidemiology, University of Washington, Seattle, WA, United States of America; 2 Department of Medicine, University of Washington, Seattle, WA, United States of America; 3 Department of Environmental and Occupational Health Sciences, University of Washington, Seattle, WA, United States of America; 4 University of Manitoba, Max Rady College of Medicine, Manitoba, Canada; 5 Department of Medicine, University of California at San Diego, San Diego, CA, United States of America; 6 Department of Medicine at the School of Medicine, University of Alabama at Birmingham, Birmingham, AL, United States of America; 7 Department of Medicine, University of California at San Francisco, San Francisco, CA, United States of America; 8 Department of Epidemiology, Gillings School of Global Public Health, University of North Carolina, Chapel Hill, NC, United States of America; 9 Center for AIDS Research, Case Western Reserve University/University Hospitals Case Medical Center, Cleveland, OH, United States of America; 10 Department of Medicine, Johns Hopkins University, Baltimore, MD, United States of America; 11 Harvard Medical School, Beth Israel Deaconess Medical Center, Fenway Health, Boston, MA, United States of America; 12 Department of Genetics and Genomic Sciences, Icahn School of Medicine at Mount Sinai, New York, NY, United States of America; 13 Public Health Sciences Division, Fred Hutchinson Cancer Research Center, Seattle, WA, United States of America; Emory University, UNITED STATES

## Abstract

**Background:**

The prevalence of substance use in people with HIV (PWH) in the United States is higher than in the general population and is an important driver of HIV-related outcomes. We sought to assess if previously identified genetic associations that contribute to substance use are also observed in a population of PWH.

**Methods:**

We performed genome-wide association studies (GWAS) of alcohol, smoking, and cannabis use phenotypes in a multi-ancestry population of 7,542 PWH from the Center for AIDS Research Network of Integrated Clinical Systems (CNICS). We conducted multi-ancestry GWAS for individuals of African (n = 3,748), Admixed American (n = 1,334), and European (n = 2,460) ancestry. Phenotype data were self-reported and collected using patient reported outcomes (PROs) and three questions from AUDIT-C, an alcohol screening tool. We analyzed nine phenotypes: 1) frequency of alcohol consumption, 2) typical number of drinks on a day when drinking alcohol, 3) frequency of five or more alcoholic drinks in a 30-day period, 4) smoking initiation, 5) smoking cessation, 6) cigarettes per day, 7) cannabis use initiation, 8) cannabis use cessation, 9) frequency of cannabis use during the previous 30 days. For each phenotype we considered a) variants previously identified as associated with a substance use trait and b) novel associations.

**Results:**

We observed evidence for effects of previously reported single nucleotide polymorphisms (SNPs) related to alcohol (rs1229984, p = 0.001), tobacco (rs11783093, p = 2.22E-4), and cannabis use (rs2875907, p = 0.005). We also report two novel loci (19p13.2, p = 1.3E-8; and 20p11.21, p = 2.1E-8) associated with cannabis use cessation.

**Conclusions:**

Our analyses contribute to understanding the genetic bases of substance use in a population with relatively higher rates of use compared to the general population.

## Introduction

Substance use is both a risk factor for acquiring HIV and a prevalent behavior among people with HIV (PWH) [1]. It is associated with poorer outcomes along the HIV Care Cascade including delayed initiation of and reduced adherence to antiretroviral therapy (ART), increased risks of HIV transmission and progression, and increased risk of adverse HIV-associated outcomes[1–5]. Over the last 25 years there has been a decline in HIV-related morbidity and mortality which has been attributed to ART allowing most PWH to live lifespans similar to those of the general population [6, 7]. While ~90% of PWH now achieve an undetectable viral load [3], substance use is one of the challenges impacting many of the 10% who have detectable viral loads.

In the general population, more than 400 genetic loci have been identified as influencing tobacco and alcohol use phenotypes, including initiation, cessation, and frequency of consumption [8]. Single nucleotide polymorphism (SNP)-based heritability estimates based on up to 1.2 million individuals range from 4% for number of alcoholic drinks per week to 8% for smoking initiation and number of cigarettes per day [8]. Substance use phenotypes have been shown to be genetically correlated [8, 9], with 60% shared heritability for lifetime cannabis and tobacco use, 36% shared heritability for lifetime cannabis and drinks per week, and 34% shared heritability for smoking initiation and drinks per week. However, previous genome-wide association studies (GWAS) of lifetime cannabis use reported only seven independent loci and a SNP-based heritability estimate of 11% [9, 10]. Additionally, previous work on the genetics of cannabis was limited to only a single binary phenotype (never/ever) and did not assess cessation or frequency of cannabis use.

Most previous research on the intersection of substance use and HIV has been conducted in relatively small samples or select groups, creating a gap in understanding the generalizability in PWH [11]. Most genetic association studies among PWH have used candidate gene approaches or focused on pharmacogenomics related to drug metabolism and adverse events. Genome-wide data in combination with comprehensive clinical and self-reported data in the Centers for AIDS Research Network of Integrated Clinical Systems (CNICS) cohort allow for investigations into the genetic contribution to outcomes relevant to the aging population of PWH.

It is important to understand the interplay between genetic predisposition, substance use, HIV, and long-term consequences of HIV infection. In this work, we describe methods for harmonizing and analyzing genotype data across a diverse population of PWH. We then utilized patient reported outcomes (PROs) collected at baseline visits in CNICS to define several substance-related phenotypes, and used these along with genome-wide genetic data to conduct genetic association studies of substance use. We compared our findings to previously published GWAS findings and identified two novel loci for cannabis-related outcomes.

## Methods

### Population: CNICS cohort

We conducted this study among PWH in the CNICS cohort [12], a well-characterized longitudinal observational cohort of PWH who enrolled in care at eight geographically distinct HIV clinics in the US from January 1995 through June 2020 at which time the data was accessed (http://www.uab.edu/cnics/). CNICS incorporates new PWH as they enter care ensuring relevance to modern HIV care. Patients are followed as part of routine clinical care. CNICS is diverse in terms of sex, self-reported race/ethnicity and geography, with 18% women, 44% White, 38% Black, and 12% Hispanic, and eight different clinic sites across the country. Reflecting an aging population of PWH, half of CNICS participants are now 50 years of age or older.

### Data: CNICS DATA repository

The CNICS data repository integrates comprehensive longitudinal data from outpatient and inpatient encounters [12]. It captures standardized HIV-related information collected at enrollment (initial clinic visit), sociodemographic, clinical, medication, and laboratory data from each site’s electronic health record and other institutional data sources. Invited participants complete the CNICS clinical assessment of PROs on touch-screen tablets at routine clinic visits every ~4–6 months [13, 14]. Participants who are medically unstable, appear intoxicated, have a cognitive impairment, or do not speak English or Spanish are not asked to complete the clinical assessment. The clinical assessment was initiated at the first site in 2007 with a new site initiating it each year or two until 2018, when all eight sites were completing clinical assessments as part of routine care visits. Most recently, Amharic has been added as an additional language and several sites also added remote options for participants completing their clinic appointments via telehealth in response to the COVID-19 pandemic. CNICS participants have completed >103,000 clinical assessments to date. The clinical assessment includes measures of smoking, alcohol, (Alcohol Use Disorders Identification Test [AUDIT/AUDIT-C] [15, 16]), and drug use (MINI International Neuropsychiatric Interview & Alcohol, Smoking and Substance Involvement Screening Test [ASSIST] [17]), and other domains.

CNICS participants were included in this study if they had genome-wide genetic data available and they had completed one or more clinical assessments. Institutional review boards at each site approved the study protocol, and all study participants provided informed consent to be included in the cohort.

#### Substance use phenotypes

Substance use information was captured using the CNICS clinical assessment of PROs. Initial assessment was used for participants who had completed the PRO multiple times.

*Alcohol use*. The AUDIT-C is a 3-item instrument on alcohol consumption and frequency designed to screen for hazardous alcohol use: We analyzed: 1) alcohol use frequency standardized to days of use in the last 30 days, 2) estimated number of drinks per week, 3) frequency of binge drinking, defined as five or more drinks on a single occasion for men and 4 or more for women, standardized to days of use in the last 30 days. All alcohol consumption analyses were restricted to participants who reported consuming alcohol (i.e., excluding those who reported never drinking).

*Tobacco use*. CNICS participants self-reported history of never, former, or current smoking and also answered items regarding duration and quantity. We analyzed three smoking phenotypes: 1) smoking initiation categorized as never versus former or current smoking; 2) smoking cessation categorized as former versus current smoking, excluding those who never smoked from analyses; and 3) among those who reported having ever smoked, the number of cigarettes per day.

*Cannabis use*. For cannabis use we analyzed: 1) cannabis use initiation, 2) cannabis use cessation, and 3) among ever cannabis users, frequency of use in the past three months standardized to days of use in the last 30 days.

For all substances, measures of frequency were recategorized as ordered factored variables (**S1A–S1I Fig**).

### Genotyping and imputation

#### Overview

**Fig 1:**
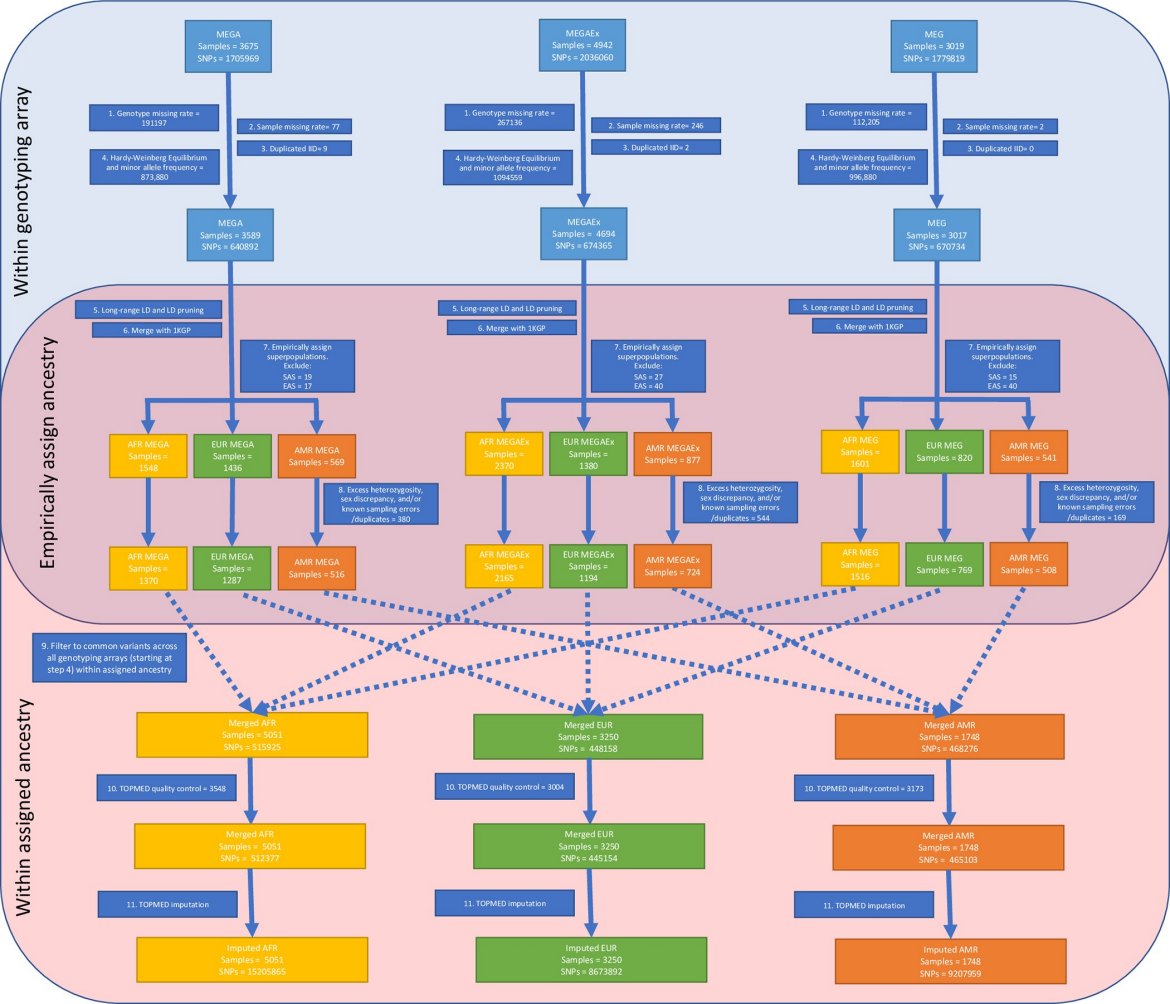
Flow diagram for sample inclusion based on genotype quality control, empirically assigned ancestry, and genotype imputation for samples with genotype data in CNICS. AFR = African; AMR = Ad Mixed American; EAS = East Asian; EUR = European; SAS = South Asian MAF = Minor allele frequency.

We applied an analysis framework for conducting GWAS in multiple ancestry populations as described in **S1 Fig** by Peterson et al. [18]. In brief, we first conducted basic quality control analyses for participants and SNPs. We then assigned each individual to a global ancestry group (African (AFR), Admixed American (AMR), East Asian (EAS), European (EUR), South Asian (SAS)) using the 1000 Genomes Project Consortium (IKGP) [19] populations as reference (see details below). Due to small sample sizes of EAS (n = 97) and SAS (n = 61), we excluded these individuals for future analyses. After additional quality control, data were then imputed using the multi-ancestry Trans-Omics for Precision Medicine (TOPMed) reference panel [20]. Details regarding number of samples and variant inclusion and exclusions are included in **Fig 1**.

#### Genotyping Arrays and quality control

Genotyping was done using the Illumina Multi-ethnic Global Array (MEGA, n = 3,675), the Expanded version (MEGAEx, n = 4,942), and Infinium Multi-Ethnic Global-8 Kit (MEG, n = 3,019). Quality control and data cleaning before imputation were done using PLINK1.9 [21]. We conducted genotyping quality control within each array by restricting to chromosome 1–22 and removing variants with a missing genotype rate ≥0.05. We then removed participants with a missing genotype rate greater than 0.05 and/or duplicated IDs. We removed variants with extreme departure from Hardy-Weinberg equilibrium (p-value<10^−30^) for variants with minor allele frequency (MAF)>0.05.

#### Empirically assigned ancestry

We removed regions of long-range linkage disequilibrium (LD) [22], and pruned for LD (window size = 10000, shift = 10, Rsq threshold = 0.1) to obtain a set of independent SNPs. We then used 1KGP to empirically assign each genotyped individual to an ancestry group [19, 23]. We identified SNPs that were included in both our pruned dataset (see above) and in 1KGP, ignoring insertions and deletions. We used the Genotype-Harmonizer to correct any allele mismatches [24]. We used smartPCA to infer principal components (PCs) based on the 1KGP population and projected these to CNICS participants [21, 25](**S1 Fig**). Within each of the 26 assigned 1KGP populations, we used the first 10 PCs to calculate the mean and covariance matrix for each 1KGP population. We then calculated the Mahalanobis distance for each subject in CNICS with all 26 1KGP populations and removed any population outliers (>4 standard deviations from the mean, 12 participants from MEGA, 14 participants from MEGAEx and no participants from MEG). We then recalculated the mean and covariance matrix for the first 10 PCs in CNICS [23], recalculated the Mahalanobis distance and assigned each individual to the closest 1KGP population based on Mahalanobis distance. Our final datasets included CNICS individuals assigned to African (AFR, n = 5,051), Admixed American (AMR, n = 1,741), and European (EUR, n = 3,250) ancestral superpopulations.

#### Ancestry-SPECIFIC quality control

Within each ancestry population and genotype array, we used the “check-sex” function in PLINK v1.9 to compute X chromosome inbreeding coefficients (parameter F) in the subset of individuals empirically assigned as AFR, AMR, or EUR. We removed pseudo-autosomal regions and excluded variants with genotype missing rate >5%, MAF<0.05. We then obtained a set of independent SNPs (r^2^<0.1) (—indep-pairwise 100kb 1 0.1). We chose an F minimum of 0.5 for female cutoff, and the default 0.8 for males after manual inspection of the distribution of F. Individuals with mismatches for reported birth sex and genetically inferred sex were removed (n = 212).

#### Merged genotype data

We merged remaining participants across genotyping arrays within ancestry based on Genome Reference Consortium Human Build 37, restricting to common SNPs between platforms using Genotype-Harmonizer to check for strand flips [24]. We used PLINK to generate PCs using the same pruning steps as described above and tested all SNPs for associations with platform (MEGAEx = 1 vs MEGA = 0) as the outcome, adjusting for 10 PCs. Significantly associated SNPs (p-value<5E-08) were removed before imputation. We repeated this step again to merge MEG to MEGA and MEGAEX.

#### TOPMed imputation and relatedness

Within assigned ancestry, we repeated our quality control (QC) pipelines, removing variants with missing genotype rates >5%, then samples with missing genotype rates >5%, restricting to chromosomes 1–22 and MAF>0.01, and removing SNPs with departure from Hardy-Weinberg equilibrium at p<10E^-8^. Data were prepared for imputation using the TOPMed imputation server at Michigan according to recommended guidelines [26] with parameter settings for TopMed and “Mixed” population reference genome GRCh38, without filtering.

We used the SNPRelate package in R to convert the TOPMed imputed VFC files to GDS formats for analyses. We included genotyped SNPs with MAF>0.05 and missingness<0.10 for calculating the genetic relationship matrix from GCTA [27]. We removed regions of long-range LD and performed LD pruning in PLINK to obtain a set of independent (r^2^<0.1) SNPs. We then calculated relatedness and PCs within ancestry groups.

### Statistical analysis

We performed genome-wide analyses within each of the three superpopulations (AFR, AMR, EUR) for the nine substance use phenotypes collected from PROs: (1) Alcohol use in the last 30 days; (2) Number of drinks on a typical day of drinking; (3) Frequency of binge drinking (≥5 drinks for men and ≥4 for women) in the last 30 days; (4) Smoking initiation; (5) Smoking cessation; (6) cigarettes per day among PWH who smoke; (7) Cannabis use initiation; (8) Cannabis use cessation; and (9) Cannabis use frequency in the last three months. We included variants with imputation quality greater than 0.8, MAF greater than 1%, and Hardy-Weinberg equilibrium p-value>10^−10^. We conducted association analyses using the GENESIS package in R [28, 29]. We created a null model using Gaussian or logistic regression, depending on outcome, by regressing the outcome on the following covariates: age at visit, first five PCs, array (indicator variable) and the genetic relatedness matrix modeled as a random effect following the GENESIS pipeline. We then performed single variant association tests assuming an additive inheritance model on the fitted null model.

We performed Meta-Regression of Multi-Ethnic Genetic Association across the three ancestry groups using MR-MEGA [30]. MR-MEGA uses genome-wide metrics of genetic heterogeneity between populations to model population variation via multi-dimensional scaling [21]. Final results were filtered to MAF>0.01 in the overall study population. We assessed genomic inflation factor (λ) and visually inspected the QQ-plots to identify statistical inflation and present the plots in **S3A–S3I Fig**. We created regional association plots using LocusZoom [31] for signals that reached genome-wide significance in the multi-ancestry GWAS. Due to low statistical power, we do not present ancestry specific GWAS results.

We compared results for alcohol use and smoking behavior to previously reported findings from Liu et al. *Nature* 2019 [8] which included up to 1.2 million individuals. For cannabis phenotypes we compared results to findings published by Pasman et al. *Nat Neurosci*. 2018 [10] which analyzed lifetime cannabis use among 184,765 individuals. We highlight previously reported SNPs with p<0.01 in CNICS as credible evidence of consistent findings which included as few as 8 known SNPs for cannabis initiation to as many as 350 for smoking initiation.

## Results

After QC exclusions, our final genetic data included 10,049 PWH, with 5,051, 1,748, and 3,250 empirically assigned to AFR, AMR, and EUR populations, respectively. Among these, 7,542 individuals had PRO data available for phenotyping and were included in our GWAS (**Table 1**). We had data on alcohol consumption for 7,252 individuals, smoking information for 7,542 individuals and information about cannabis use for 7,280 individuals. The proportion who reported ever smoking varied by assigned ancestry, with 70% of those of EUR ancestry reporting either current or former smoking compared to 62% of AFR ancestry and 57% of AMR ancestry. Among those with cannabis use information, 74% of those of EUR ancestry reported cannabis use in the last year compared to 61% of AMR and 56% of those assigned to AFR ancestry.

**Table 1:** Sample sizes for substance use related phenotypes among samples in CNICS within genetically assigned ancestry.

	AFR (N = 5,051)	AMR (N = 1,748)	EUR (N = 3,250)	Total (N = 10,049)
**Alcohol** *	(n = 3,477)	(n = 1,307)	(n = 2,460)	(n = 7,244)
1) Alcoholic drinks per week	2,144	889	1,679	4,712
2) Number of drinks	2,139	888	1,674	4,701
3) Binge drinking	2,140	887	1,678	4,705
**Smoking**				
4) Never vs. ever	3,748	1,334	2,460	7,542
5) Current vs. former	2,304	762	1,719	4,785
6) Smoking frequency	2,282	753	1,710	4,745
**Cannabis**				
7) Never vs. ever	3,553	1,319	2,408	7,280
8) Current vs. former	1,995	801	1,780	4,576
9) Cannabis frequency	938	364	768	2,070

*Note that alcohol data was available for max n and analyses were then subset to those who reported any drinking. Measures of current versus former and frequency of smoking and cannabis use were subset to ever users.

### Alcohol

We compared our genetic association results to those previously reported by Liu et al. [8]. Of 99 variants reported by Liu et al. to be associated with drinks per week, a previously reported non-synonymous variant in the Alcohol Dehydrogenase 1B (*ADH1B)* gene was nominally associated with drinks per week in CNICS and directionally consistent (rs1229984, p = 0.001, MAF = 0.03, ß_Liu_ = 0.15, ß_CNICS_ = 0.43) (**Table 2**; **S1 Table in S1 Data**). However, no variants reached p<0.01 for frequency of drinking in the last 30 days or for frequency of binge drinking in our multi-ancestry analysis (**S2, S3 Tables in S1 Data**).

**Table 2:** Strongest associations for substance use phenotypes among PWH in the U.S. among previously published SNPs.

	Chromosome:Position:Ref:Alt		rsID		Published p-value	CNICS p-value	Annotation
**Alcohol**							
Number of drinks	chr4:99318162:T:C		rs1229984		<2.2E-308	0.001	Nonsynonymous:*ADH1B*
**Smoking**							
Never vs. ever	chr1:18110163:G:T		rs3820277		1.57E-13	0.005	*IGSF21*/NM_032880:+:Intron
	chr1:95948779:T:G		rs1935571		6.99E-10	0.005	Intergenic
	chr2:103510525:A:G		rs1901477		2.07E-31	0.004	Intergenic
	chr5:30841947:T:G		rs12517438		1.89E-09	0.007	Intergenic
	chr6:41934025:C:T		rs3218116		1.05E-11	0.006	Intergenic
	chr8:27567832:C:T		rs11783093		2.07E-41	0.0002	Intergenic
	chr8:27568560:T:A		rs1565735		1.33E-09	0.008	Intergenic
	chr10:104700702:T:C		rs9787523		1.42E-09	0.003	*SORCS3*/NM_014978:+:Intron
	chr16:69542991:A:G		rs9302604		3.29E-13	0.006	Intergenic
Current vs. former	chr8:27568560:T:A		rs1565735		1.54E-12	0.008	Intergenic
	chr19:40847202:T:C		rs56113850		1.61E-48	0.007	*CYP2A6*/NM_000762:-:Intron

Direction of effects were all consistent with previously published SNPs

### Smoking

Among 351 SNPs reported by Liu et al. to be associated with smoking initiation at a genome-wide significant level, nine showed associations at p<0.01 with smoking initiation in our multi-ancestry analysis (**Table 2; S4 Table in S1 Data**), with the strongest association for rs11783093 (8p21.1, p = 2.22E-4, MAF = 0.08, ß_Liu_ = -0.05, ß_CNICS_ = -0.23). For smoking cessation, of the 21 SNPs reported by Liu et al. overlapping with our data, two SNPs were associated at p <0.01, including rs56113850 in the *CYP2A6* gene (**Table 2; S5 Table in S1 Data**). Among the 51 SNPs associated with cigarettes per day among people who smoke reported by Liu et al, none reached p<0.01 in our multi-ancestry results (**S6 Table in S1 Data**). Direction of effects were also consistent with published beta coefficients for all SNPs which reached p<0.01 in our analyses.

### Cannabis

We extracted the results for seven SNPs previously reported by Pasman et al. to be associated with lifetime cannabis use [10]. None of the seven SNPs was associated with cannabis use phenotypes at p<0.01 in our multi-ancestry analysis (**S7-S9 Tables in S1 Data**). In ancestry-specific analyses, one SNP located in *CADM2* showed nominal association with cannabis initiation (rs2875907, p = 0.005, MAF = 0.36, ß_Pasman_ = 0.07, ß_CNICS_ = 0.20) in the EUR only GWAS for which the direction of effect was consistent with the beta estimate found by Pasman et al. (**S7 Table in S1 Data**). We did not observe any replication of previous findings for cannabis cessation (**S8 Table in S1 Data**). One SNP at 17p13.3 near the *SMG6* gene was nominally associated with frequency of cannabis use standardized to the last 30 days (rs17761723, p = 3.97E-4, MAF = 0.07, ß_Pasman_ = 0.05, ß_CNICS_ = 0.69) in the AFR only GWAS with the same direction of effect for the alternative allele (**S9 Table in S1 Data**).

In the multi-ancestry GWAS of cannabis cessation, we observed two novel genome-wide significant associations at 19p13.2 (rs311780, p = 1.29E-8, MAF = 0.19) and 20p11.21 (rs6113974, p = 2.07E-8, MAF = 0.04) (**S4A and S4B Fig; Table 3**). The lead SNP at locus 19p13.2 showed no evidence of association in the AMR population (ß = 0.03, MAF = 0.06, p = 0.91), but modest effects in EUR (ß = -0.45, MAF = 0.05, p = 0.0045) and in AFR where this variant was more common (ß = -0.36, MAF = 0.36, p = 2.58E-7). Conversely, for locus 20p11.21, we observed associations in EUR (ß = 0.85, MAF = 0.04, p = 8.08E-7) and in AFR (ß = 0.48, MAF = 0.05, p = 0.003), but no evidence in the AMR population (ß = 0.35, MAF = 0.03, p = 0.20).

**Table 3:** Independent genome-wide significant loci for cannabis use cessation in meta-analysis of PWH across ancestries.

		MR-MEGA (N = 4,576)		AFR (N = 1,995)			AMR (N = 801)			EUR (N = 1780)		
Chromosome:Position	rsID	Beta (SE)	P-value	MAF	Beta (SE)	P-value	MAF	Beta (SE)	P-value	MAF	Beta (SE)	P-value
chr19:11460095:G:A	rs311780	-0.35 (0.07)	1.29E-8	0.36	-0.36 (0.07)	2.58E-7	0.06	-0.027 (0.24)	0.910	0.05	-0.45 (0.16)	4.51E-3
chr20:23169112:G:A	rs6113974	0.60 (0.14)	2.07E-8	0.05	0.48 (0.16)	2.64E-3	0.03	0.35 (0.27)	0.203	0.04	0.85 (0.17)	8.08E-7

AFR = African

AMR = Admixed American

EUR = European

SE = standard error

MAF = minor allele frequency

## Discussion

In this study, we set out to assess if known genetic associations for substance use (alcohol, smoking and cannabis) are also observed in a diverse population of PWH living in the US. One of the most well-known genetic associations with alcohol consumption is the non-synonymous variant rs1229984 located in the *ADH1B* gene [8]. We observed an association of this variant with the number of drinks on a typical day of drinking (p = 0.001), as well as with the frequency of drinking in the last 30 days in individuals of admixed American ancestry (p = 0.001). When comparing our results to the largest GWAS of smoking (conducted in 1.2 million individuals, Liu et al.) we found evidence of the important role of the nicotine metabolism gene *CYP2A6* in relation to both smoking cessation and cigarettes per day [8]. However, we only observed support for the published SNP in the EUR population and not in the AFR population despite the larger sample size, suggesting that perhaps another SNP may better tag the causal variant(s) in that population. Proposed mechanisms through which genetic variation may alter individual predisposition for substance use have largely involved neurotransmission, dopamine systems reward pathways, and stress response [32]. It is possible that numerous exposures in PWH alter these pathways, including immunosuppression, long-term treatments, and societal factors related to stigma and marginalization. We recognize that we are likely underpowered due to sample to detect many previously associated SNPs.

We conducted GWAS of nine substance use phenotypes for up to 7,542 PWH living in the United States. We report novel findings at two loci (19p13.2 and 20p11.21) for cessation of cannabis use, a sparsely studied phenotype in GWAS. SNPs intronic to *ELAVL3* at 19p13.2 is a neural-specific RNA-binding protein with a suggested role in neurogenesis. Differential expression of the *ELAVL3* gene has been shown in brain tissues [33]. While it has been suggested to play a role in spatial learning [34], no links have been made to substance use or behavioral traits previously. Genetics may influence cannabis cessation more than lifetime use of cannabis, which is likely a product of societal norms rather than biologic influences. There are no strong plausible candidate genes close to the 20p11.21 signal.

In the 2019 National Survey on Drug Use and Health (NSDUH), 55% of people age 18 and older in the United States reported drinking alcohol in the past month [35]. Consistent with previous work on the prevalence of substance use in PWH [36], upwards of 65% of our study population reported having consumed alcohol in the last month. That same report found that 34% of adults 18 and older report binge alcohol use in the last month. Lifetime use of tobacco products was an estimated 63% in adult 18+ in the NSDUH [35]. Smoking prevalence is known to be higher in PWH than the general population [37], and in our population, we observed a life-time prevalence of 63%. The NSDUH found 49% of people aged 18 or older reported ever using marijuana in their lifetime [35], as compared to 63% in CNICS. In the context of genetic analyses, these overall higher prevalence among PWH allow for additional investigations into factors associated with cessation as the numbers of ever users of alcohol, tobacco, and cannabis tend to be higher than in the general population, albeit to varied degrees according to ancestry.

An important strength of this study is our high-quality phenotype measurements. CNICS has served as clinical care sites for the Patient Reported Outcomes Measurement Information System (PROMIS), enabling a focus on valid measurement approaches for PROs to minimize societal bias and maximize ascertainment of substance use phenotypes. We were able to capture detailed information about alcohol use through the use of questions from a validated tool, the AUDIT-C, including assessment of both frequency and amount of alcohol consumption; two factors which are suggested to have independent influences on hazardous drinking [38]. This work represents a geographically and racially diverse population. Our genetic approach to empirically assign samples to reference ancestry populations and perform within ancestry GWAS mitigates the potential confounding due to population stratification and structure. We leverage this diversity to assess the generalizability of previous research on the genetic architecture of substance use, which predominantly relies on populations of nearly exclusive European ancestry.

We recognize that our relatively small sample sizes compared to previous work make our analyses generally underpowered to detect genome-wide significant associations for traits with low heritability. Our findings for cannabis cessation, if replicated, may suggest that cannabis cessation has a genetic component. Unfortunately, our small sample sizes limit our ability to estimate genome-wide SNP heritability of these traits. Neuronal acetylcholine nicotine receptors have long been implicated for their role in nicotine dependence [39]. In Liu et al. the strongest association was for *CHRNA3*, but we did not observe any significant effect of genetic variants in this gene. Understanding whether and to what extent genetic factors are associated with substance use patterns would be a valuable insight into helping reduce and mitigate the effects of substance use disorders in PWH.

In summary, we validate previous findings of the genetic effects of the *ADH1B* gene on alcohol consumption in a population with relatively high alcohol intake, and specifically on number of drinks on a typical day of drinking. We report novel findings from our multi-ancestry GWAS of cannabis cessation in PWH, but we stress the need for independent replication of these results. We present a methodological framework for future genetic analyses to be conducted in a diverse and underrepresented population with extensive and well characterized behavioral and clinical data.

## Supporting information

S1 Fig Distributions of nine substance use phenotypes included in GWAS analyses for PLWH. (DOCX)

S2 Fig Principle components of merged CNICS participants with 1KGP eigenvalues for overlapping SNPs. (DOCX)

S3 Fig MR-MEGA QQ plots for nine substance use GWAS in PLWH. (DOCX)

S4 Fig LocusZoom plots for genome-wide significant signals from the multi-ancestry GWAS of cannabis use cessation in PLWH in the United States. (DOCX)

S1 Data Replication of GWAS findings from for substance use related phenotypes in PLWH within and across ancestries. (XLSX)
