# Supplementary material for: Assessing the associations between known genetic variants and substance use in people with HIV in the United States
Source: PLoS One. 2023 Oct 5;18(10):e0292068. doi: 10.1371/journal.pone.0292068 (PMC10553320; doi:10.1371/journal.pone.0292068)
Supplement: S1 Fig — (DOCX) [file pone.0292068.s001.docx]

| **Supplementary Figures 1A-I**: Distributions of nine substance use phenotypes included in GWAS analyses for PLWH. | | |
| --- | --- | --- |
| A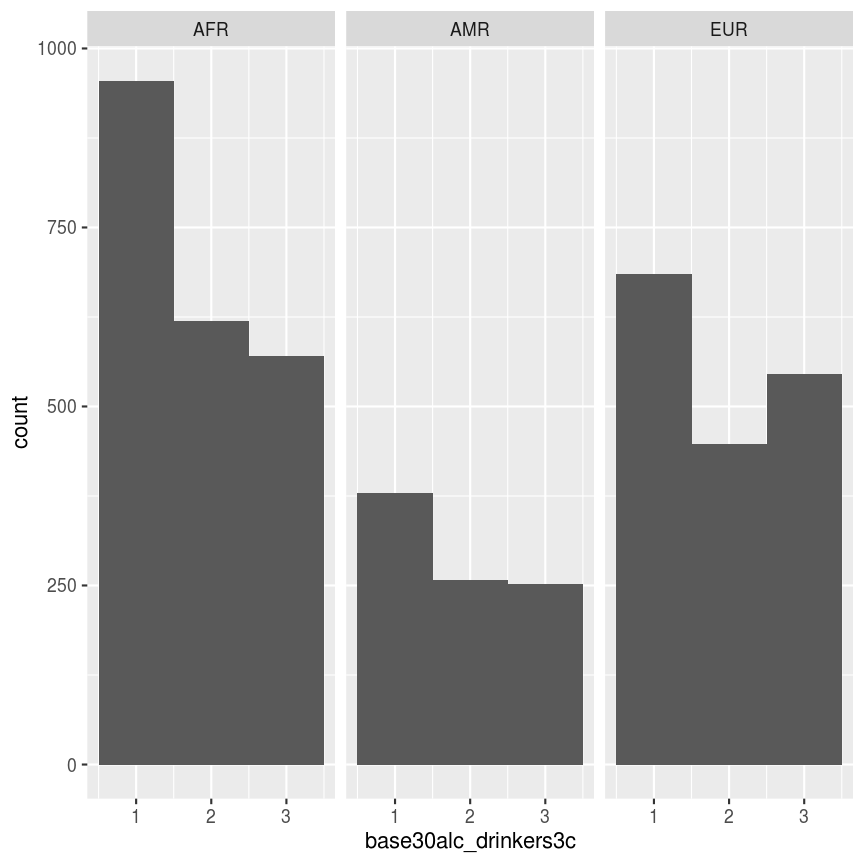 | B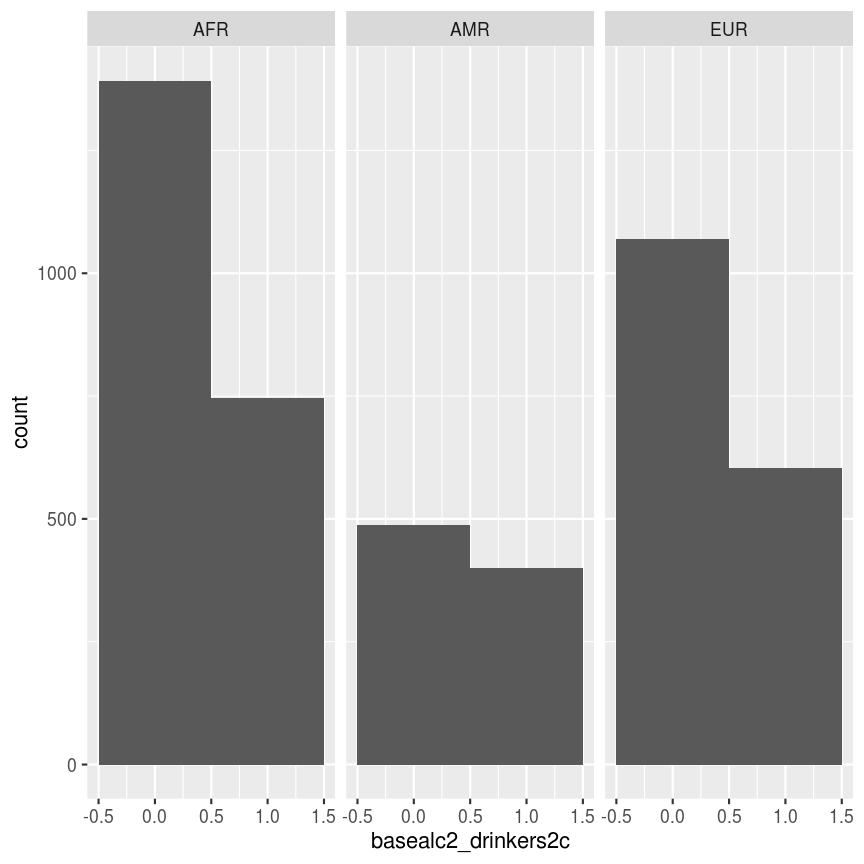 | C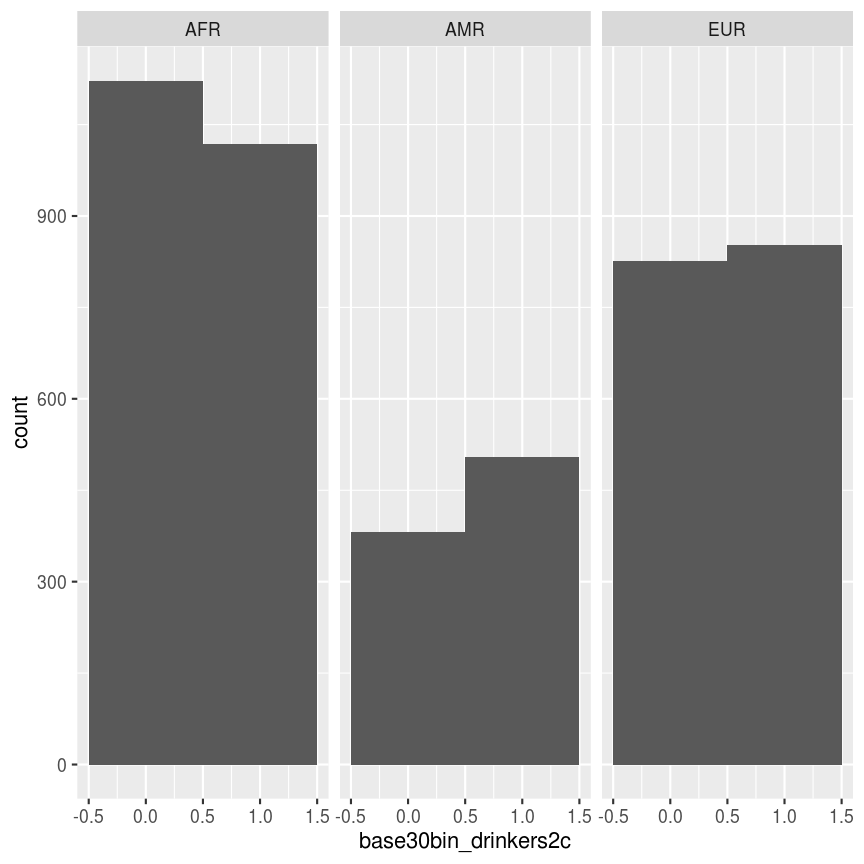 |
| D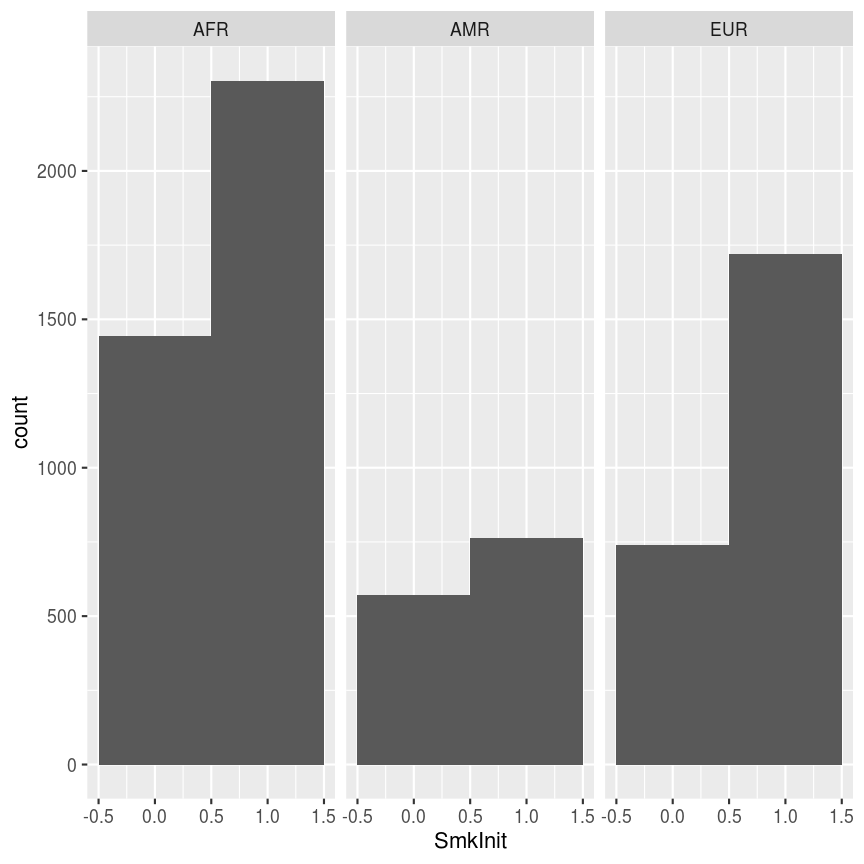 | E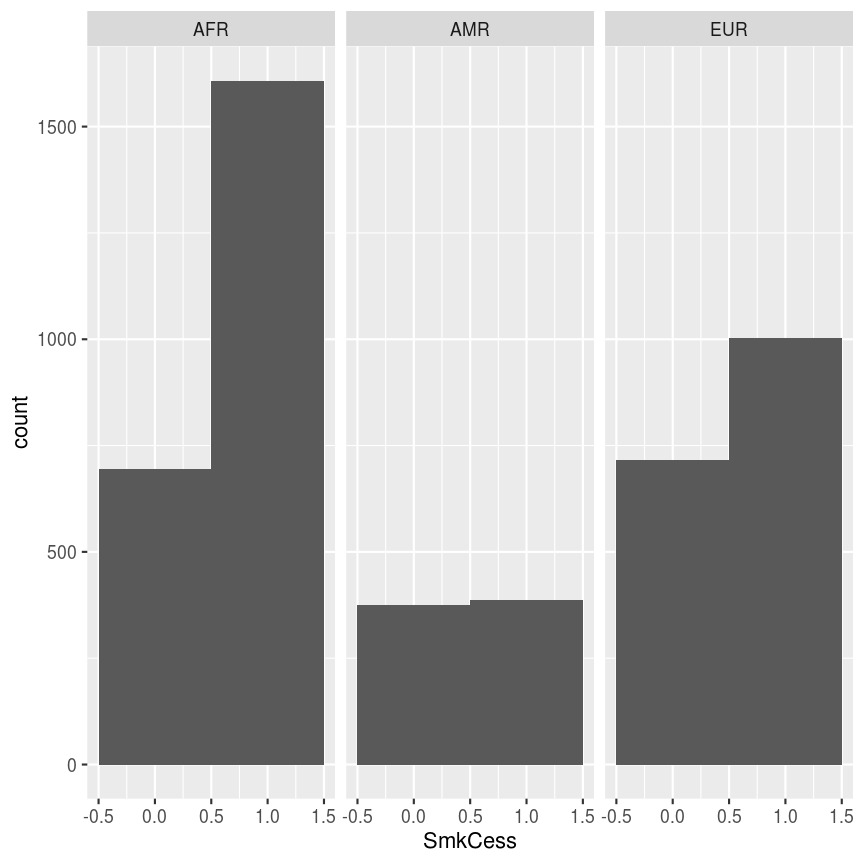 | F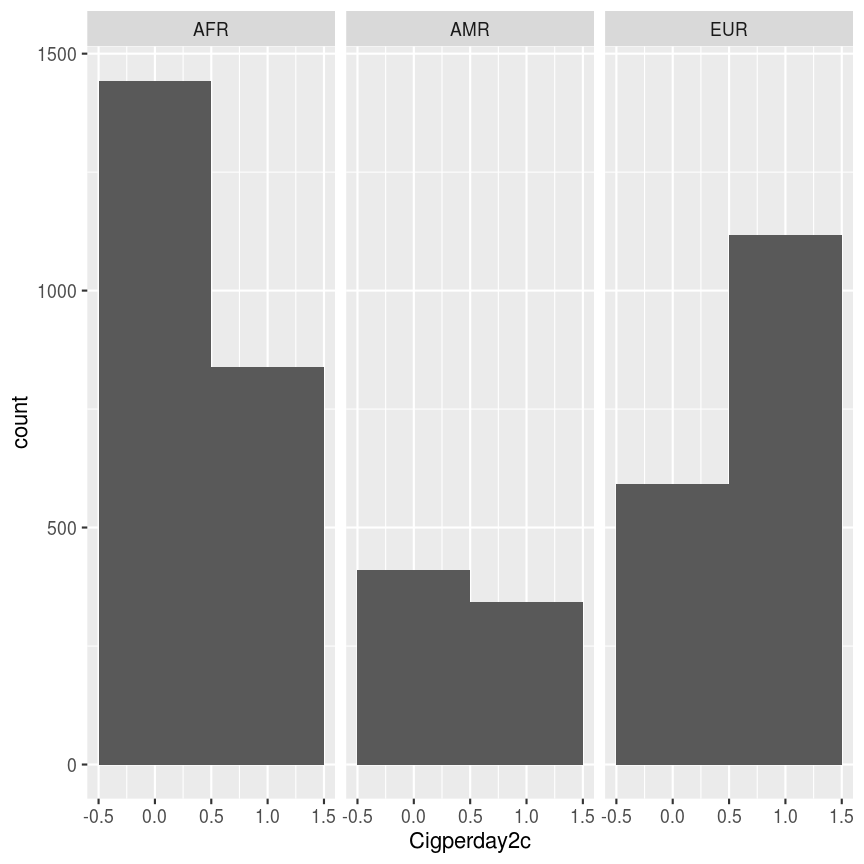 |
| G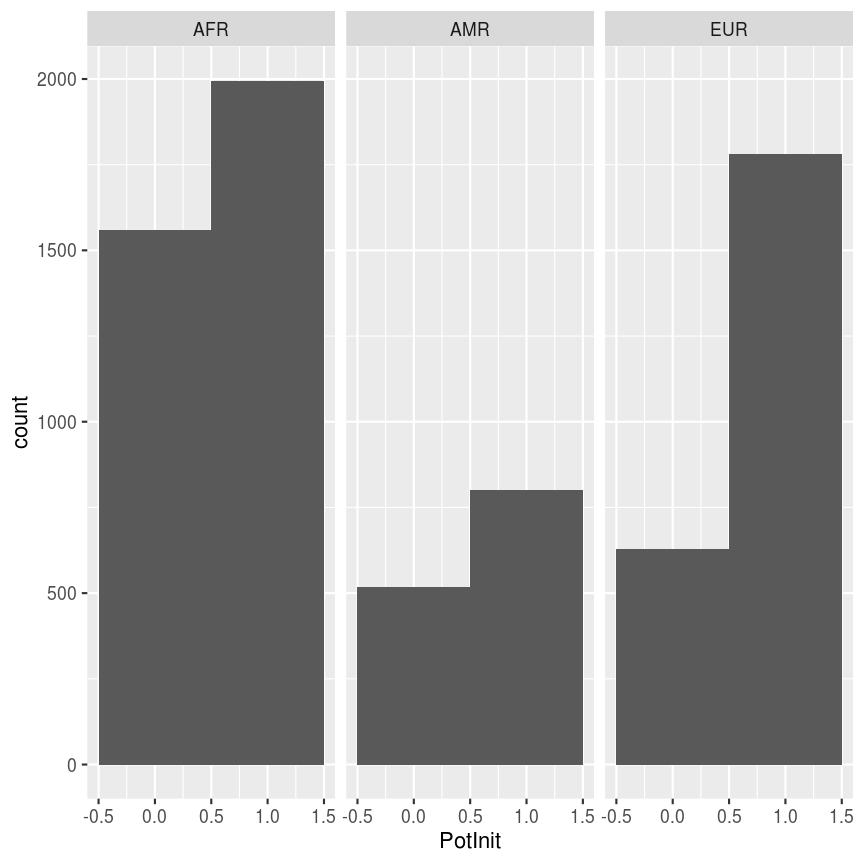 | H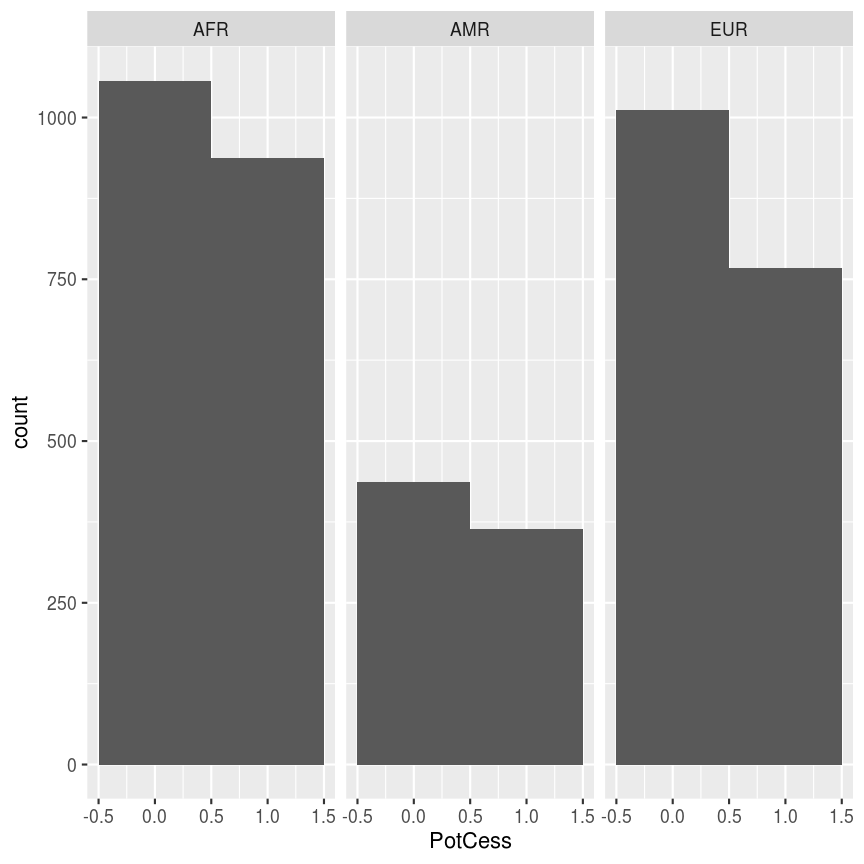 | I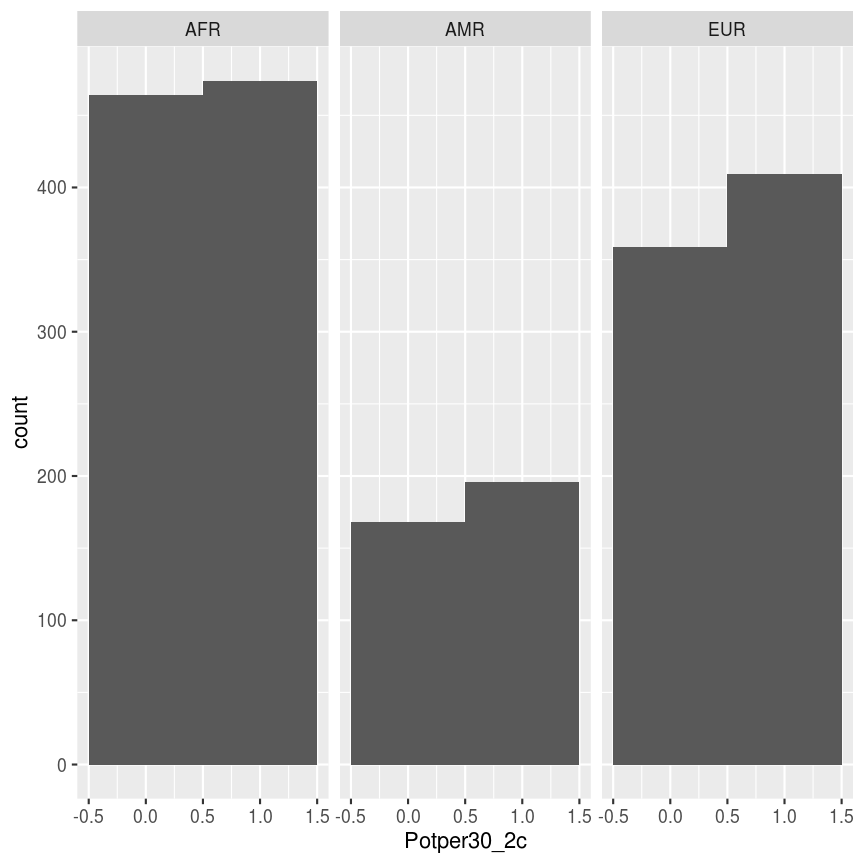 |
| (A) Question 1 of the AUDIT-C: Alcohol use in the last 30 days - continuous; (B) Question 2 of the AUDIT-C: Number of drinks on a typical day of drinking - binary; (C) Question 3 of AUDIT-C: Frequency of binge drinking (5+ drinks) in the last 30 days - binary; (D) Smoking initiation - binary; (E) Smoking cessation - binary; (F) cigarettes per day among smokers - continuous; (G) Cannabis use initiation - binary; (H) Cannabis use cessation - binary; and (I) Cannabis use frequency in the last three months - binary. | | |
