# Supplementary material for: Assessing the associations between known genetic variants and substance use in people with HIV in the United States
Source: PLoS One. 2023 Oct 5;18(10):e0292068. doi: 10.1371/journal.pone.0292068 (PMC10553320; doi:10.1371/journal.pone.0292068)
Supplement: S2 Fig — (DOCX) [file pone.0292068.s002.docx]

| **Supplementary Figure 2**. Principle components of merged CNICS participants with 1KGP eigenvalues for overlapping SNPs. |
| --- |
| 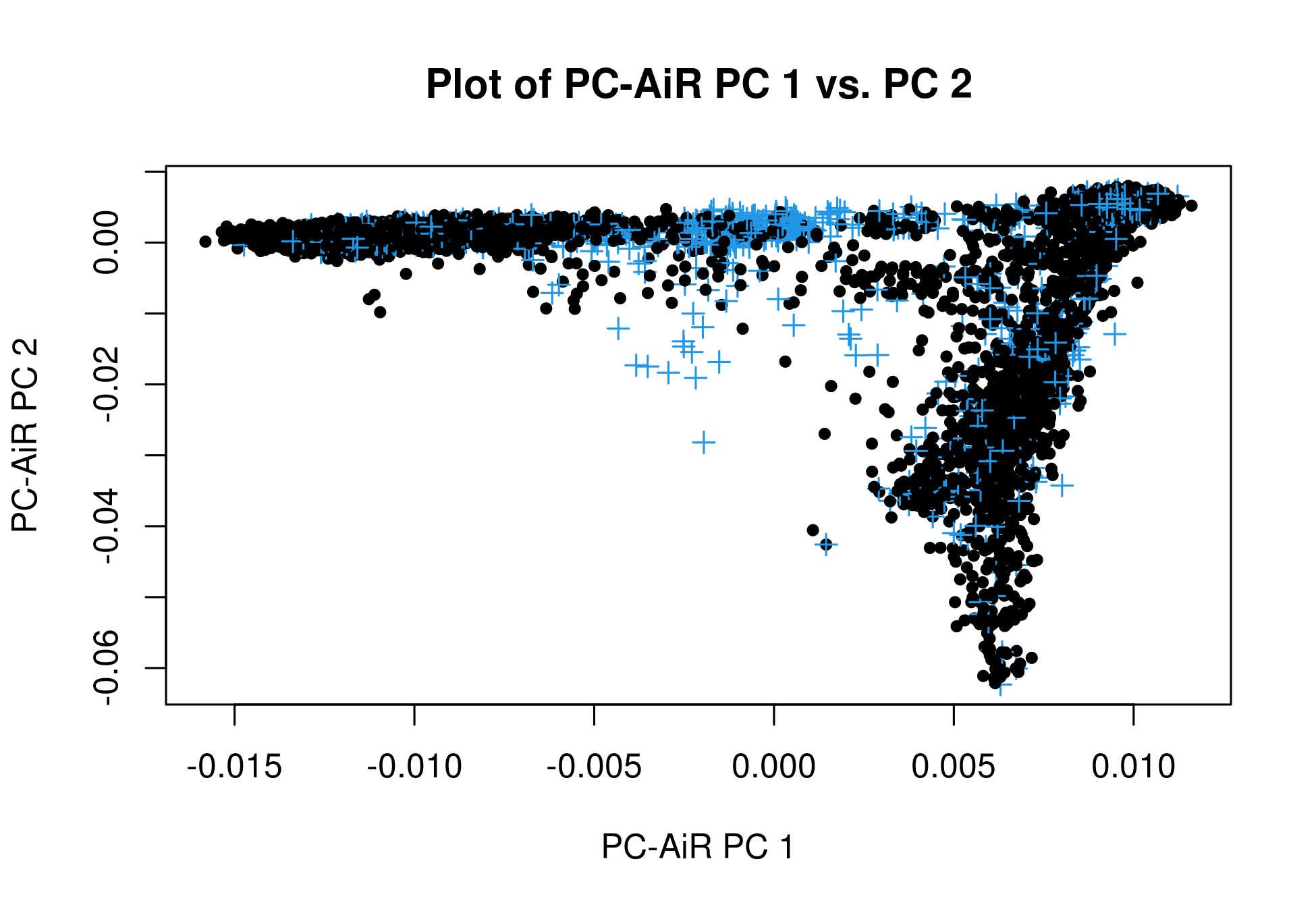 |
| CNICS = Center for AIDS Research (CFAR) Network of Integrated Clinical Systems  1KGP = 1000 Genomes Project (reference population indicated by blue +)  SNP = single nucleotide polymorphism |
