# Supplementary material for: Assessing the associations between known genetic variants and substance use in people with HIV in the United States
Source: PLoS One. 2023 Oct 5;18(10):e0292068. doi: 10.1371/journal.pone.0292068 (PMC10553320; doi:10.1371/journal.pone.0292068)
Supplement: S3 Fig — (DOCX) [file pone.0292068.s003.docx]

| **Supplementary Figures 3A-I**: MR-MEGA QQ plots for nine substance use GWAS in PLWH. | | |
| --- | --- | --- |
| A  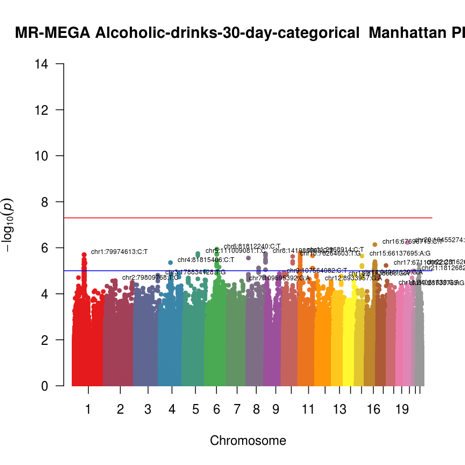  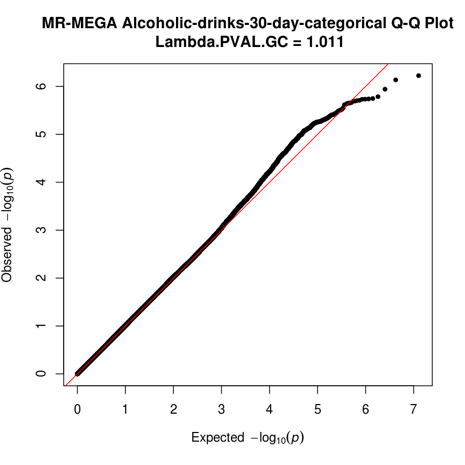 | B  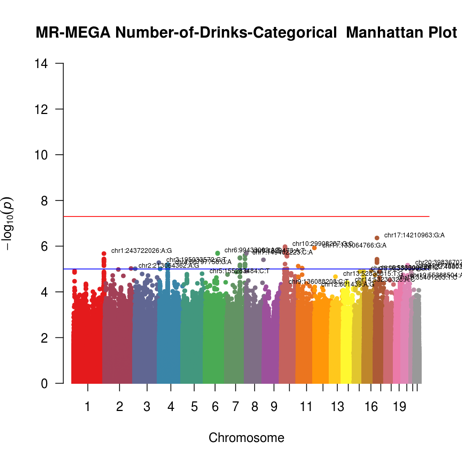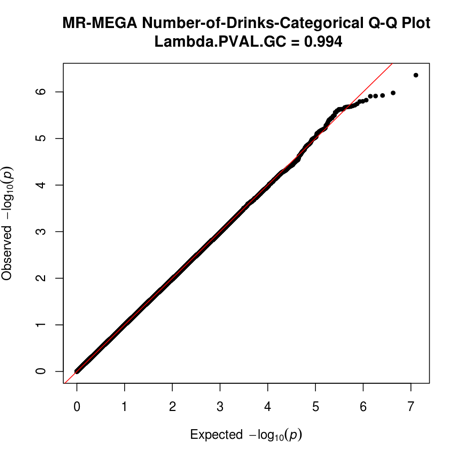 | C  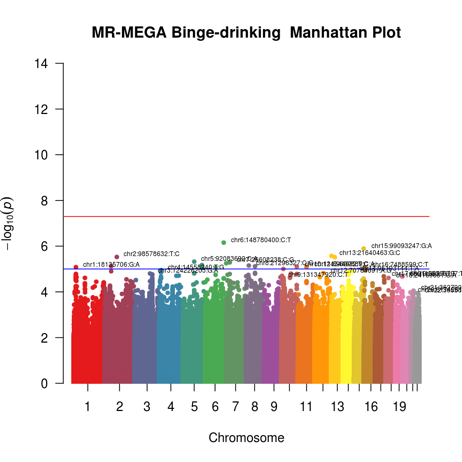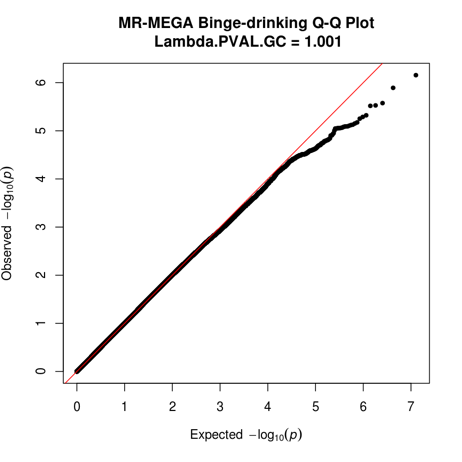 |
| D  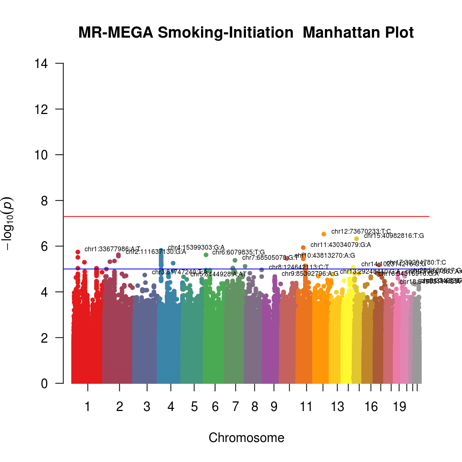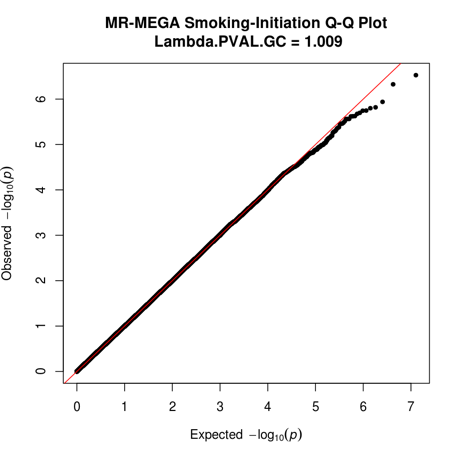 | E  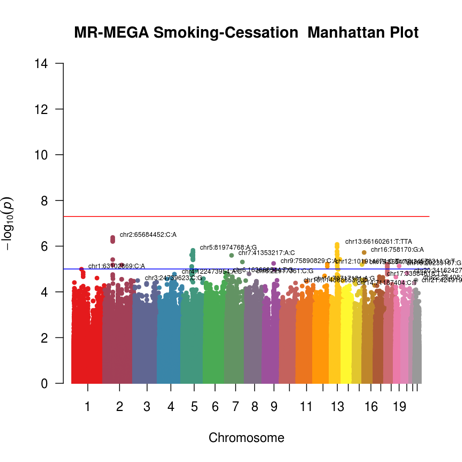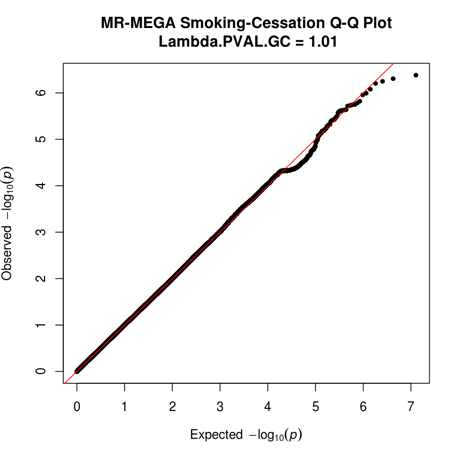 | F  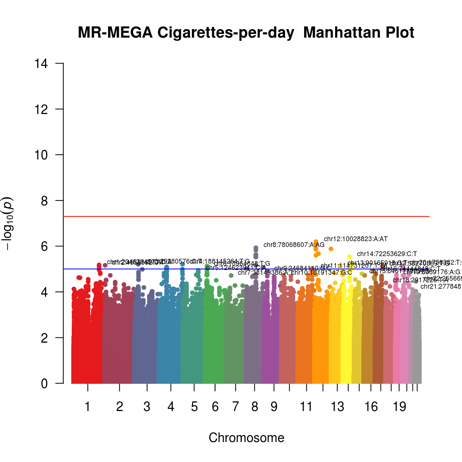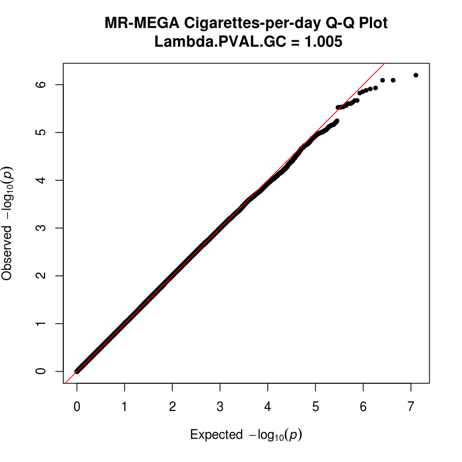 |
| G  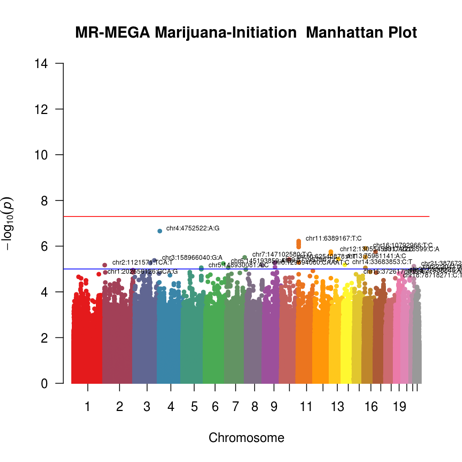  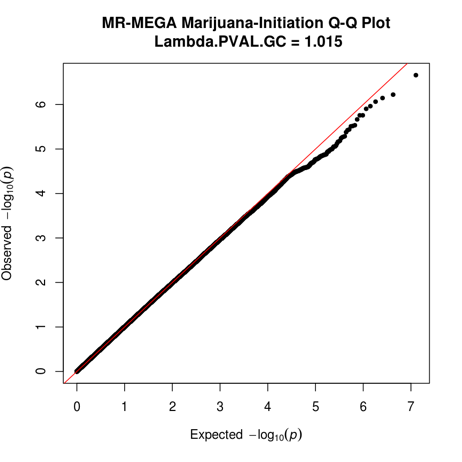 | H  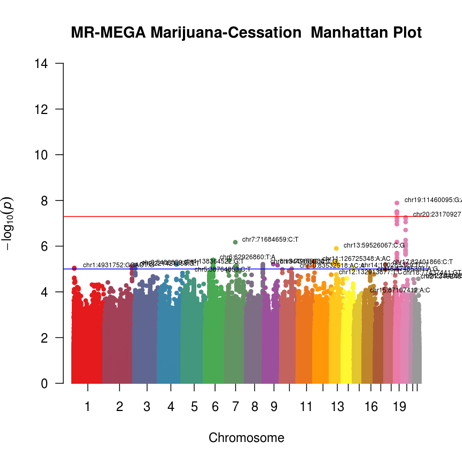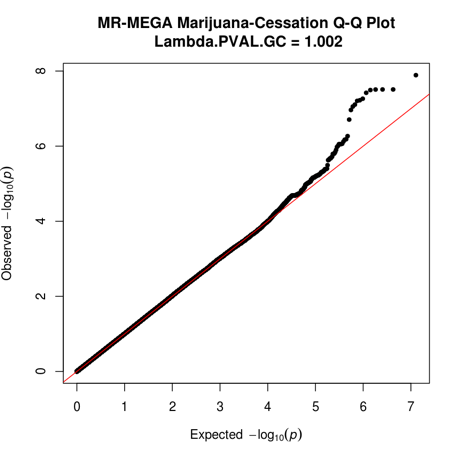 | I  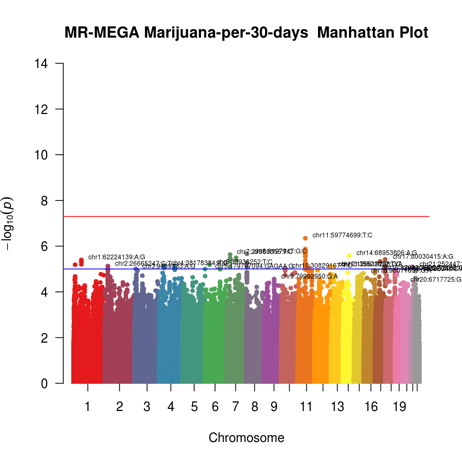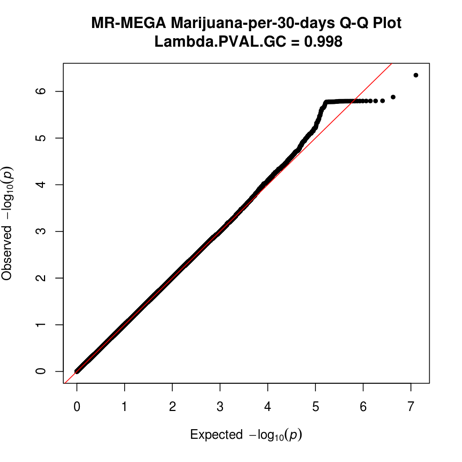 |
| (A) Question 1 of the AUDIT-C: Alcohol use in the last 30 days; (B) Question 2 of the AUDIT-C: Number of drinks on a typical day of drinking; (C) Question 3 of AUDIT-C: Frequency of binge drinking (5+ drinks) in the last 30 days; (D) Smoking initiation; (E) Smoking cessation; (F) cigarettes per day among smokers; (G) Cannabis use initiation; (H) Cannabis use cessation; and (I) Cannabis use frequency in the last three months. | | |
