# Supplementary material for: Assessing the associations between known genetic variants and substance use in people with HIV in the United States
Source: PLoS One. 2023 Oct 5;18(10):e0292068. doi: 10.1371/journal.pone.0292068 (PMC10553320; doi:10.1371/journal.pone.0292068)
Supplement: S4 Fig — (DOCX) [file pone.0292068.s004.docx]

| **Supplementary Figures 4A-B**: LocusZoom plots for genome-wide significant signals from the multi-ancestry GWAS of cannabis use cessation in PLWH in the United States. |
| --- |
| AB |
